# Advance social information allows red crossbills (*Loxia curvirostra*) to better conserve body mass and intestinal mass during food stress

**DOI:** 10.1098/rspb.2022.0516

**Published:** 2022-05-25

**Authors:** J. M. Cornelius

**Affiliations:** Integrative Biology, Oregon State University, Corvallis, OR 97331, USA

**Keywords:** metabolism, behaviour, bird, phenotypic plasticity, gut flexibility

## Abstract

Animals prepare for fluctuations in resources through advance storage of energy, planned reduction in energy costs or by moving elsewhere. Unpredictable fluctuations in food, however, may be particularly challenging if animals cannot avoid negative impacts on body condition. Social information may help animals to cope with unpredictable resources if cues from individuals with low foraging success give advance warning about deteriorating conditions. This study investigates the impact of social information on behaviour and physiology of food-restricted captive red crossbills (*Loxia curvirostra*). Birds were restricted to two short feeding periods per day to simulate a decline in resources and were given social information from food-restricted neighbours either before (i.e. predictive) or during (i.e. parallel) the food-restriction period. Focal birds better conserved body mass during food restriction if social information was predictive of the decline in resources. Crossbills with predictive information ate more food, had larger intestinal mass and better conserved pectoral muscle size at the end of the restriction period compared to those with parallel social information. These data suggest that birds can use social information to alter behavioural and physiological responses during food shortage in ways that may confer an adaptive advantage for survival.

## 1. Introduction

Animals employ all manner of strategies to cope with challenging environments, ranging from seasonal avoidance strategies like hibernation or migration, to coping strategies like caching or altered foraging behaviour [1]. Physiological adjustments in metabolic rate, digestive capacity and energy reserves may accompany behavioural changes, but may require some preparation and take time or resources to execute [2–5]. Unpredictable environmental conditions may therefore be particularly challenging for many animals. Sudden storms, droughts, food shortages or other habitat disturbances can cause high mortality of adults and offspring [6–9], thus coping mechanisms for such disturbances are probably under strong selection in many environments on earth.

Animals gather information about their environments through individual experience but may also use public information gathered through interactions with or observations of other individuals [10]. Public information can improve the accuracy of environmental assessment [11,12], help individuals find rare or novel food resources [13–15] and help to coordinate group responses, although it may also come with a cost if public information is sometimes inaccurate [16]. Public information may be particularly important during unpredictable environmental change when recent historical knowledge of an environment is mismatched with changing conditions [17–19]. If public information can provide predictive information in regard to impending environmental challenges, then it may also improve fitness by allowing for preparations that reduce the physiological repercussions of environmental stress. The benefits of predictive social information have been explored in prey species that produce acoustic, visual or chemical warning signals in response to the detection of a predator [20,21], but public information about declining food could also help individuals to respond earlier or more strongly to resource challenges.

The value of public information can increase if food becomes unpredictably distributed and individual search costs increase [17]. Public information can help animals to locate patchily distributed resources, but animals must also handle the food, digest the food and assimilate the desirable nutrients in the gut [22]. Digestion can be rate-limiting for energy intake, as is the case in herbivores foraging on lower quality food items [23,24]. Digestive efficiency (i.e. assimilation) is dependent on the nutritive quality of the food items and on many physiological factors, including enzyme dynamics, transporter density, gut transit time and gut size [25–29]. Animals can flexibly regulate many of these properties to improve assimilation, but improving assimilation (i.e. building a larger gut or increasing enzyme and transporter densities) may require resources and time, and also come at the cost of a heavier, more metabolically expensive gut [3,30–32]. Such trade-offs are perhaps most apparent in organisms with costly modes of locomotion, such as in birds requiring flight [33,34].

The red crossbill (*Loxia curvirostra*) is a nomadic songbird that copes with unpredictable food resources and is responsive to public information in the context of food [11,35,36]. Prior research has found that the presence of a food-restricted neighbour enhances the secretion of the stress hormone corticosterone during a food stressor [35] and reduces corticosteroid receptor expression in control regions of the brain [37]*.* This reduction in corticosteroid receptors may sensitize the hypothalamic–pituitary–adrenal axis to stressors, thereby preparing the bird to respond more strongly to an impending challenge [37–40]. I hypothesize that predictive social information from food-restricted neighbours will induce coping mechanisms that prepare birds for challenging food conditions. Specifically, I predict that red crossbills with predictive social information will better conserve body mass during time-limited foraging trials through increased food intake, build-up of fat reserves and preservation of intestinal mass. Further, given that food challenge in the wild may require increased searching behaviour or—in the worst-case scenario—escape migrations to an alternative habitat [41–43], I also predict protection of the main muscle for flight locomotion (i.e. the pectoral muscle) during food restriction in those birds with predictive social information.

## 2. Material and methods

**Figure 1.**
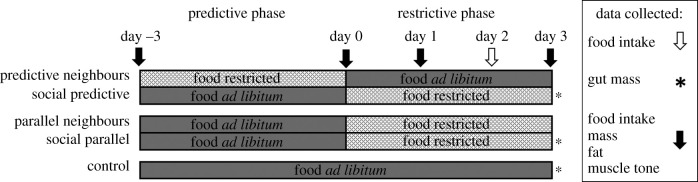
Experimental timeline.

This experiment was organized into a preparative phase during which focal birds received social information from well-fed or restricted neighbours, followed by a restrictive phase during which focal birds were food restricted. Focal birds were provided with social information from food-restricted neighbours for either 3 days prior to their food restriction (i.e. social predictive focal group) or for 3 days coincident with the focal food restriction (i.e. social parallel focal group; figure 1). The total amount of social information received about declining food was thus similar between the two focal groups; however, this design also resulted in different social information contexts during the restrictive phase: predictive focal birds had well-fed neighbours whereas parallel focal birds had restricted neighbours (figure 1). To control for this, I also compare the response to food restriction in the neighbour groups. Neither of the neighbour groups received predictive information prior to their respective restrictions; however, the predictive neighbours were housed next to well-fed birds during their restriction whereas the parallel neighbours were housed next to restricted birds (figure 1; electronic supplementary material, table S1 for summary).

### (a) Capture and captive conditions

The red crossbill species is a complex of different eco-types that are morphologically adapted to particular species of conifer seeds and which can be acoustically identified by type-specific vocalizations [36,44]. This experiment uses type 2 red crossbills, which are found frequently in ponderosa pine (*Pinus ponderosa*)-dominant habitats [45]. Type 2 red crossbills (*n* = 96) were captured via mist net at various field sites in the east Cascade mountain range of Oregon and Washington, USA in the late summer of 2019. Birds were held in captive facilities at Oregon State University on natural photoperiod and room temperature, and with *ad libitum* pellet food and daily sunflower seed treats until the experiment started the following summer. All birds were handled once per week for a separate experiment that monitored seasonal changes in body condition from March to early June. A subset of birds (*n* = 24) experienced a long-day photoperiod exposure for several weeks in late March (see electronic supplementary material for details). These birds were split equally between the predictive neighbour and parallel neighbour groups and we detected no differences in initial mass or in their response to food restriction compared to individuals that had not previously been photoadvanced (electronic supplementary material, figure S1).

### (b) Social information

Sex and age-at-capture were balanced across all treatment groups in three experimental rooms (electronic supplementary material, table S2). Focal birds were housed alone in a small cage (42 cm L × 30 cm W × 56 cm H) with visual contact with one pair of neighbours housed in a cage next to them. Neighbours were the same sex as the focal bird and included one juvenile and one adult in each pair. Control individuals were visually separated from other birds but could hear the other control birds. Similarly, each social group could hear the other individuals of their treatment group only (i.e. one treatment group per experimental room).

### (c) Food restriction and food intake

Birds were fed a commercial maintenance pellet food designed for songbirds (see electronic supplementary material for nutritional information). Controls had access to food *ad libitum* for the entire experiment. Focal and neighbour birds in the social groups each experienced 3 days of food restriction during which they had access to food for two 45 min feeding sessions per day [19,46]. Food was provided in a covered cup to minimize spills and food intake was calculated as the change in grams of food remaining in the cup across a 24 h period, measured with an electronic balance to the nearest 0.01 g. Multiple food cups were provided in neighbour cages to ensure both birds had access to food during the feeding periods. One bird in the social predictive group regularly flung food out of its cage, thus food intake was not calculated for this bird.

### (d) Body condition metrics

Body mass was measured with an electronic balance to the nearest 0.01 g. Muscle and fat deposit scores were collected by a single experienced observer to the nearest 0.25 scale unit in a partially blind design. The observer knew the stage of the experiment and from which room data were being collected, but did not know the bird's individual identification or if the bird was from a neighbour or focal group at the time of scoring. Pectoral muscle size was scored on a scale of 1 to 4 based on the shape of the muscle around the keel, described in detail by Bairlein [47]. Fat deposits were scored in the abdominal and furcular cavities based on Helms & Drury [48] and described in detail for red crossbills by the author previously [41].

### (e) Organ mass and carcass lipid composition

Six randomly chosen individuals from the control, social predictive and social parallel groups were euthanized by an overdose of isoflurane, plucked free of feathers and immediately dissected for wet organ mass measurements immediately following the day 3 sampling session. Another six individuals from the social parallel group were sacrificed later after having been refed on *ad libitum* food for two weeks to determine how intestinal mass in food-restricted birds responded to refeeding and to help interpret the observed patterns during restriction. I did not sample from all groups to reduce the number of birds sacrificed. Wet masses of liver, gizzard, oesophagus, stomach, intestine and colon were collected using an electronic balance to the nearest hundredth of a gram. Alimentary organs were each cleared of contents by cutting each structure open with surgical scissors, rinsing with water and then blotting dry prior to measurement. Organs were immediately placed back in the carcass and the carcass was frozen at −80°C. Percentage lipid in dried carcass mass was determined by Soxhlet extraction (see electronic supplementary material for details) to test if visual scoring of subcutaneous fat stores predicted whole-body lipid composition.

### (f) Activity

Activity was monitored continuously throughout the experiment with infrared activity monitors from STARR Life Sciences (Oakmont, PA, USA). These monitors record the number of beam breaks over time and capture whole-body movements using an array of infrared beams projected throughout the cage. Sensor ID and position on the cage were kept constant for each individual throughout the course of the experiment to minimize any impact of detection variability on changes in activity levels. Monitor position ensured that activity was captured from only a single focal cage. Three sensors failed during the experiment (two in the social predictive and one in the social parallel).

### (g) Statistical analysis

Analyses were performed separately for the predictive and restrictive phases of the experiment. The analysis for mass, fat and muscle included all treatment groups; however, neighbour groups were excluded from activity and food intake analyses because they were housed in pairs and it was not possible to assign measures to an individual. Parallel neighbour and social parallel groups had similar timing and type of social information (electronic supplementary material, table S1) and were not different in any of the measured variables across sample days (Tukey *p* > 0.3 for mass, fat or muscle), thus these two groups were combined to simplify analyses and graphical representations.

Data were tested for normality and all dependent variables were analysed using linear mixed models (LMMs) with the exception of muscle size data, which were analysed using non-parametric comparison of means tests because transformations failed to normalize the distribution of raw data. Activity data were log transformed. LMMs for body mass, fat deposit, food intake and activity were fit by restricted maximum likelihood and included treatment and the interaction between treatment and sample day as fixed effects. Age and sex and their interactions with sample day and treatment group were initially included as fixed effects in all models but were removed due to a lack of significance (*p* > 0.15) and the *a priori* expectation that responses would be similar in these groups [35,37]. Individual identification was included as a random effect in all models. Significant fixed effects were further explored by model parameter estimates and Tukey comparisons of means. Effect tests for LMMs are presented in table 1 and parameter estimates are presented in the electronic supplementary material, tables S3–S10. *Post hoc* comparisons of means are reported in figure legends and text.

**Table 1.** Effect tests for LMMs describing changes in food intake, activity, mass and fat before (*a*) and during (*b*) food restriction. *F*-statistic, DF, DFDen and *p*-values shown for fixed effects. Parameter estimates are provided in the electronic supplementary material.

	food intake			activity		mass		fat	
(*a*) predictive phase									
sample day	*F*_1,32_ = 0.2		*p* = 0.64	*F*_1,34_ = 2.3	*p* = 0.14	*F*_1,89_ = 2.2	*p* = 0.13	*F*_1,90_ = 2.5	*p* = 0.12
treatment	*F*_2,34_ = 2.2		*p* = 0.13	*F*_1,34_ = 2.0	*p* = 0.14	*F*_3,90_ = 4.9	*p = 0.003*	*F*_3,3_ = 12.6	*p* < 0.0001
treatment by day	*F*_2,32_ = 4.4		*p* = 0.02	*F*_1,34_ = 4.3	*p* = 0.02	*F*_3,90_ = 7.8	*p* = 0.04	*F*_3,3_ = 1.4	*p* = 0.24
individual ID	*p* = 0.0002			*p* < 0.0001		*p* < 0.0001		*p* < 0.0001	
(*b*) restrictive phase									
sample day	*F*_1,75_ = 23	*p* < 0.0001		*F*_2,71_ = 36	*p* < 0.0001	*F*_1,183_ = 411	*p* < 0.0001	*F*_1,183_ = 320	*p* < 0.0001
treatment	*F*_2,35_ = 73	*p* < 0.0001		*F*_2,35_ = 3.8	*p* = 0.03	*F*_3,90_ = 2.2	*p* = 0.10	*F*_4,89_ = 4.6	*p* = 0.002
treatment by day	*F*_2,75_ = 6.7	*p* = 0.002		*F*_2,71_ = 10.7	*p* < 0.0001	*F*_3,183_ = 73	*p* < 0.0001	*F*_4,183_ = 24	*p* < 0.0001
individual ID	*p* < 0.0001			*p* < 0.0001		*p* < 0.0001		*p* < 0.0001	

Changes in muscle size were analysed by non-parametric Wilcoxon comparison of means for sex and age or Kruskal–Wallis rank sums for treatment group. The relationships between the change in body mass and changes in fat, muscle or intestinal mass were explored using linear regression, and intestinal mass was compared across social treatment groups with ANOVA followed by a one-tailed *t*-test for *a priori* directional predictions (i.e. predictive versus parallel social groups) and two-tailed *t*-test for comparisons with the control group. To validate visual fat scoring metrics, we compare the percentage fat of dry mass determined by Soxhlet extraction (*n* = 24) to visual fat scores using linear regression, with the exclusion of a single outlier in visible fat stores (greater than 2 SD from mean), and between treatment groups using a Student's *t*-test.

## 3. Results

### (a) Predictive phase

#### (i) Behaviour

There were no independent effects of sample day or treatment on either food intake or activity, but there was a marginal interactive effect of treatment by sample day on both food intake (*p* = 0.02) and activity (*p* = 0.02; table 1*a*). Parameter estimates (electronic supplementary material, tables S3 and S4) suggest that the change in food intake and activity in the social predictive group was significantly different from the control group during the predictive phase; however, the beta estimates are small (food intake *β* = 0.17; activity *β* = 0.03) and *post hoc* comparisons of means could find no differences between groups or across sample dates (electronic supplementary material, figures S2 and S3). No differences were detected in food intake or activity levels between the social predictive and social parallel groups during the predictive phase.

#### (ii) Physiology

**Figure 2.**
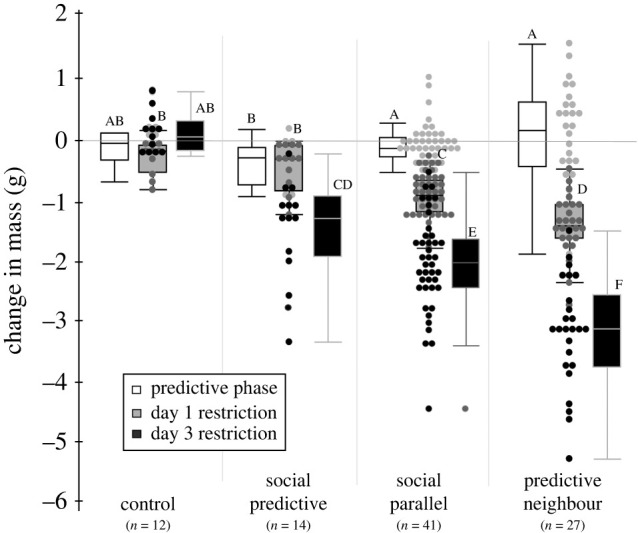
Change in body mass during time-limited foraging in captive red crossbills with different schedules of social information. Changes in mass during the predictive phase of the experiment (white bars) did not differ from the control, but red crossbills with predictive social information lost less mass than those with parallel social information after 1 day (medium grey) and 3 days (dark grey) of food restriction. Predictive neighbours, with no predictive or parallel social information, lost the most mass during restriction. Boxes denote the interquartile range and whiskers show minimum and maximum. Letters denote groups with significantly difference changes in mass by Tukey comparison of means (*p* < 0.05).

Body mass was predicted by treatment (*p* = 0.003) and a treatment × sample day interaction (*p* = 0.04; table 1*a*). The treatment effect was driven by a lighter starting mass of the control group (*β* = −2.21) and higher starting mass of the predictive neighbour group (*β* = 1.30) relative to the social predictive group (electronic supplementary material, table S5). There was no difference in starting mass between the social predictive and the social parallel focal groups. The model detected a weak interactive effect between sample day and treatment (*p* = 0.04) during the predictive phase, but parameter effect comparisons with the social predictive group revealed only a very weak potential difference with the predictive neighbours (*β* = 0.03, *p* = 0.06; electronic supplementary material, table S5). Post hoc comparisons suggest the social predictive group may have lost more mass during the predictive phase compared to the other social groups—but it was not different from controls (Tukey *p* < 0.05; figure 2). Fat deposits varied by treatment group (*p* < 0.0001) because controls had smaller fat deposits (*β* = −1.34). Fat deposit did not vary by sample day and there was no interaction effect between sample day and treatment during the predictive phase of the experiment (table 1*a*; electronic supplementary material, table S6 and figure S3A). Muscle condition was not different between any treatment groups or across sample days in the predictive phase of the experiment (Kruskal–Wallis *p* > 0.50).

### (b) Restrictive phase

#### (i) Behaviour

Sample day, treatment group and the interaction between treatment and sample day significantly predicted food intake during the restrictive phase (table 1*b*). Control birds ate more food than either of the restricted groups (*β* = 1.79; Tukey *p* < 0.0001), but restricted birds with predictive social information ate slightly more food during restriction than did birds with parallel social information (*β* = −0.92; electronic supplementary material, figure S2 and table S7)—probably because they increased food intake between the first and third day of restriction (Tukey *p* < 0.05), whereas birds with parallel information had not (Tukey *p* = 0.26).

Sample day, treatment and the interaction between sample day and treatment each contributed to activity levels (table 1*b*). Control birds were generally more active than either social group (*β* = 0.29, *p* = 0.01; electronic supplementary material, figure S3 and table S8). Food-restricted birds reduced their activity levels as food restriction progressed whereas controls did not, with a significant decline occurring in restricted birds between days 1 and 2 of restriction (electronic supplementary material, figure S3; table S8; treatment × day interaction *p* < 0.0001 and *post hoc* matched pair analysis *p* < 0.05). The change in activity after 24 h of restriction may have been slightly different between the two social groups: birds with predictive social information slightly decreased activity whereas birds with parallel social information slightly increased activity (Tukey *p* < 0.05), but the interaction was not detected in the model (electronic supplementary material, table S8).

#### (ii) Physiology

Sample day and the interaction between treatment and sample day significantly predicted mass during food restriction (table 1*b*). Birds with predictive information lost more mass during the food restriction compared to the control group (*β* = 0.59, *p* < 0.0001), but less mass compared to either the social parallel (*β* = −0.13, *p* = 0.0005) or the predictive neighbour (*β* = −0.54, *p* < 0.0001) groups during restriction (electronic supplementary material, table S9; figure S2). *Post hoc* Tukey tests suggest that the mass loss in the predictive group was smaller than the social parallel group on both days 1 and 3 of food restriction (figure 2; Tukey *p* < 0.05).

**Figure 3.**
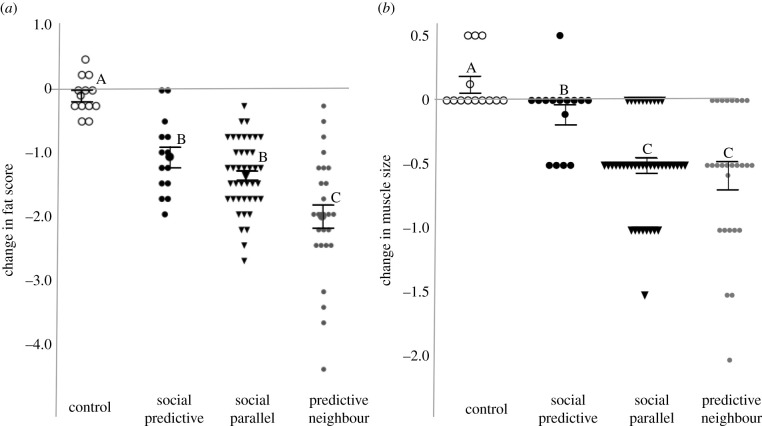
Changes in fat deposit and muscle size after 3 days of food restriction in red crossbills with different schedules of social information. Birds with predictive information (large black circle) lost similar amounts of fat (*a*) but better conserved muscle size (*b*) during food restriction relative to birds with parallel social information (black triangle). Well-fed controls (open circle) showed minimal change in either parameter and birds with no predictive information and with well-fed neighbours during restriction showed the largest changes (Predictive neighbour, small grey circle). Whiskers show mean changes ± s.e.m. Group averages with different letters are significantly different (fat: Tukey *p* < 0.05; muscle: Wilcoxon each pair *p* < 0.05).

Food restriction also caused declines in fat deposits and muscle size, but patterns varied in degree of change between social groups. Controls carried less fat than did restricted birds to start the experiment but showed no change across sample day (electronic supplementary material, table S10; figure 3*a*; Tukey *p* > 0.05). Birds with predictive information lost more fat during the food restriction than did the control group (*β* = 0.35, *p* < 0.0001), but less fat than the predictive neighbour group (*β* = −0.30, *p <* 0.0001; figure 3*a*; electronic supplementary material, table S10). There was also a weak difference detected in the model between the social predictive and social parallel groups fat deposits across sample day (*β* = −0.07, *p* = 0.02; electronic supplementary material, table S10), suggesting that the social predictive group lost less fat than the social parallel group; however, post hoc Tukey tests could not detect a difference in the change in fat between these two groups (figure 3*a*; Tukey *p* = 0.51). The percentage lipid in dry carcass mass collected after 3 days of food restriction correlated positively with visible fat stores at the time of sacrifice (*F*_1,10_ = 22.2, *r*^2^ = 0.71, *p* = 0.001) and did not differ between restricted birds with predictive or parallel social information (*t*_10_ = 0.29, *p* = 0.77).

#### (iii) Muscle size

Change in muscle size did not differ by sex or age within any treatment group or sample interval (Wilcoxon *p* > 0.05), except for in the predictive neighbour group: juveniles and adults had a similar change in muscle size after 24 h of food restriction but juveniles had a smaller change in muscle size than did adults after 72 h ($\bar{x}=-0.8$ in adults and −0.2 in.juveniles; $\chi_{1,25}^{2}=8.6$, *p* = 0.003). Kruskal–Wallis rank sums test suggests that the change in muscle size after food restriction was different between treatment groups ($\chi_{3,93}^{2}=29.3$, *p* < 0.0001). Control birds showed no change in muscle size across sample days and Wilcoxon comparisons of means suggest that all food-restricted groups showed reduced muscle size relative to the controls (social parallel *Z* = −4.5, *p* < 0.0001; predictive neighbour *Z* = −3.9, *p* < 0.0001), but only marginally so in the social predictive group (*Z* = −2.1, *p* = 0.04). The social predictive group maintained a larger muscle size during food restriction compared to both the social parallel (*Z* = 3.3, *p* = 0.001) and predictive neighbour (*Z* = 2.8, *p* = 0.005) groups (figure 3*b*).

#### (iv) Body mass change versus fat and muscle

Changes in fat deposits and muscle size strongly predicted the change in body mass after 3 days of food restriction in red crossbills (linear regression; fat *F*_1,93_ = 99.8, *R*^2^ = 0.5, *p* < 0.0001; muscle *F*_1,93_ = 37.9, *R*^2^ = 0.3, *p* < 0.0001).

#### (v) Digestive organ mass

**Figure 4.**
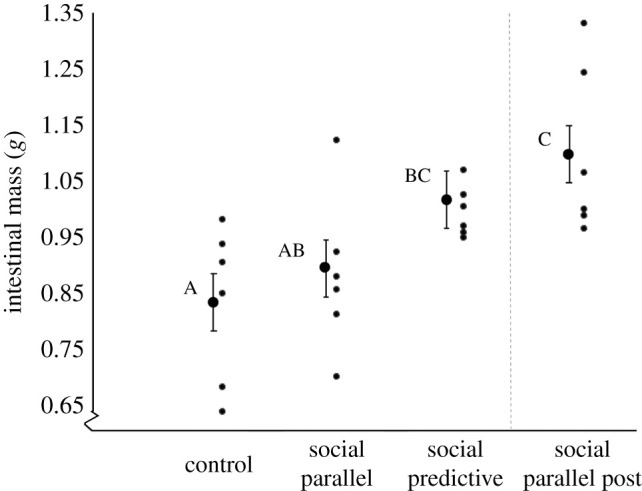
Intestinal mass following food restriction in captive red crossbills with different schedules of social information. Birds with predictive social information had larger intestines after 3 days of food restriction compared to birds with parallel social information or well-fed controls (left panel). Birds with parallel information that were fed unlimited food for two weeks after the restriction (social parallel post; right panel) had large intestines that were similar in size to restricted birds with predictive information. Whiskers show mean changes ± s.e.m. and group averages with different letters are significantly different (Student's *t*; *p* < 0.05).

Liver, oesophagus, gizzard, stomach and colon mass did not vary across treatment groups at the time of sacrifice (*p* > 0.10). Intestinal mass was significantly different across treatment groups (ANOVA *F*_3,23_ = 6.1; *p* = 0.004), but did not vary by age (*p* = 0.98), sex (*p* = 0.95) or body size (*p* = 0.44). Birds with predictive social information had larger intestines than birds with parallel information (one-tailed *t*-test *p* = 0.03) and controls (two-tailed *t*-test, *p* = 0.01; figure 4). Food-restricted birds with parallel social information did not differ in intestinal mass from well-fed controls immediately after restriction (two-tailed *t*-test; *p* = 0.39), but developed larger intestines after two weeks of *ad libitum* access to food (two-tailed *t*-test; *p* < 0.05). The intestinal mass of these recovered birds was statistically indistinguishable from that of the food-restricted birds with predictive information (two-tailed *t*-test; *p* = 0.26; figure 4). Intestinal mass did not, however, predict the observed changes in body mass during food restriction when all birds were combined (linear regression *F*_1,11_ = 0.14, *p* = 0.71).

## 4. Discussion

Birds given social information about declining food did not prepare for impending food challenge by increasing mass or changing behaviour prior to food restriction; however, social information did improve the physiological outcomes during food restriction and had the most protective effect on body condition if the information was received by captive red crossbills in advance of the decline in food. This suggests that birds can respond to social information about declining food resources to better cope with impending or current food challenges. Such responses could be variable in nature. In this experiment, birds that received predictive social information about declining food ate slightly more during time-restricted feedings and may have reduced activity faster and maintained higher intestinal mass—which probably led to higher nutrient assimilation during food restriction and lower energy costs [49]. Social information may thus improve the chances of survival during times of food shortage by allowing for physiological and behavioural adjustments.

The mechanisms that underlie the effects of social information were not explored in the current study—though prior research suggests that changes in receptor expression in the HPA axis may play a role [37]. Of particular interest is the possibility of interplay between the HPA and the gut, which has been explored extensively in mammals but less so in birds [50,51]. Birds with predictive information in this study maintained larger intestines, which may have contributed to higher assimilation rates and helped birds to limit mass loss during food restriction. Intestinal morphology is a highly flexible trait in many birds and can be regulated adaptively in response to changing conditions or behavioural states [52]. For example, migratory birds allow the gut to atrophy during long-distance flight which confers the simultaneous benefit of providing nutrients to working muscles and reducing flight costs by reducing the mass of an unused organ [3,29,34]. However, refuelling requires that the gut be rebuilt over the course of several days before the bird can efficiently gain mass [29,31,32]. Environmental changes that limit food may make it difficult to maintain or build assimilation capacity, thus compounding the consequences of lower food consumption with lower nutrient assimilation. While mild food restrictions can induce some species to grow larger intestines (e.g. *Gallus gallus domesticus* [53]), more severe restrictions cause mass loss in the intestines. A reduction of 66% or 70% in *ad libitum* food intake in yellow-rumped warblers (*Dendroica coronata*) and blackcaps (*Sylvia atricapella*), respectively, caused intestinal mass to decline by 20% and 30% [54,55]. Red crossbills in this study were reduced by approximately 70% of their normal intake for 3 days, and the social parallel group had 13% smaller intestines than did the social predictive group at the end of restriction. Surprisingly, the intestinal mass of the well-fed control group was also small and was not different from the food-restricted social parallel group, suggesting that the social predictive group may have grown larger intestines either before or during food restriction. The control group, however, consumed slightly less food, maintained a lighter body mass and was more active compared to the other groups—perhaps due to their visual isolation from other birds. Further, refeeding of the social parallel group caused a 25% increase in intestinal mass to match that of the social predictive group during restriction—thus, it is also possible that intestinal mass atrophied during food restriction but did so to a lesser degree in the social predictive group. Predictive social information therefore either triggered gut hypertrophy before the food-restriction period or prevented gut atrophy during the food-restrictive period. Either way, the birds with predictive social information had larger intestines at the end of the 3-day food restriction, suggesting that social information altered gut dynamics during restriction and may have protected assimilation capacity.

A reduction in food intake can cause metabolic imbalance and there are multiple endogenous stores of energy from which this deficit could be remedied in birds, including subcutaneous fat deposits stored in adipocytes, intracellular fat stores in various other types of cells (e.g. intestine, liver and muscle) and body protein stores [56]. Which type of store a bird accesses to provide nutrients during a fast is dependent on the size of these stores, the behavioural state of the bird and the duration of the catabolic state [49]. Migrant birds begin long-distance flights using glycogen catabolism but quickly switch to intensive fat catabolism after they have had time to initiate specialized fat transport and processing mechanisms [3,57]. Some protein may also be catabolized to provide amino acids for gluconeogenesis and fatty acid oxidation during long-distance flights, but most of the proteins used for these purposes come from alimentary organs unless energy reserves run low [58]. The reliance on lipids and targeted protein catabolism of the gut allows for lightweight fuelling of energy-intensive flight while also preserving the flight muscles [59]. Birds facing extreme food reductions may need to make irruptive movements to escape deteriorating conditions [43], thus it may be advantageous to protect muscle stores and maximize fat catabolism to maintain mobility. All food-restricted birds in our study lost fat stores during food restriction but only those with predictive social information were able to maintain pectoral muscle size. This suggests that predictive social information altered the metabolic strategy used by birds during food restriction such that muscle condition was preserved.

The observed behavioural adjustments to food restriction were slight between social information groups, but in concert, they may have contributed to the preservation of body mass observed in birds with predictive social information. Activity levels declined over time in all food-restricted birds—regardless of social information delivery—suggesting that the time-limited feedings imposed a relatively severe energy deficit. Many animal taxa show increased activity during mild or short-term food restriction [19,60–62] but then reduce activity if restriction more severely limits energy intake over time [49,60]. In this study, there was an interaction that suggested that birds with predictive social information may have initiated this decline in activity slightly earlier than birds with parallel social information. If so, the difference was subtle—as were differences in food intake. Food intake during the preparative phase of the experiment and after 1 day of restriction was very similar between those with predictive information and those with parallel information—thus predictive social information did not appear to have primed the birds for hyperphagia. However, after 3 days of restriction the birds with predictive information were eating about a half a gram more each day. It is unknown if this difference reflects the gut plasticity that was observed in response to social information (i.e. a larger gut allowed for the higher food intake) [25,32], or if the difference reflects other undescribed neural processes enacted during food restriction that altered feeding behaviour [55].

The timing of social information from restricted birds and the type of social information during the food restriction both appeared to impact the responses to food restriction in this study. The predictive neighbour group had no social information about declining food either before or during their restriction and this group showed the largest loss of mass, driven by large reductions in both fat and muscle. Prior studies have found that food-restricted crossbills paired with a well-fed bird have a smaller corticosterone response than do those that are paired with another restricted bird [35]. The authors hypothesized that a larger HPA response may help birds to better cope with food shortages by inducing changes in behaviour and physiology, but they could not fully address this hypothesis because social information was only provided in parallel (i.e. there was no predictive information available). Activity levels also trended higher in past studies in birds that were restricted in parallel, which may have driven a larger loss of mass and fat deposits in these groups [35,37]. These results stand in apparent contrast to this study where birds reduced activity during restriction and those with restricted neighbours better conserved body mass compared to those with well-fed neighbours. However, there are important distinctions. First, the food restriction in this study was much more severe (i.e. 70% restriction compared to 25% restriction previously), which could drive the reduced activity and higher mass loss. Alternatively, the observed differences may be related to the behavioural ecology and underlying physiology of the populations of crossbills used in the two studies. Red crossbills occur as a suite of different ecological types that each specialize on different groups of conifer trees [63,64]. The type 3 crossbills used in prior studies may be more prone to food-related migratory movements than the type 2 crossbills used in this experiment [41], which may in part underlie the difference in activity caused by food restriction in the studies—though both types are nomadic and deal with fluctuations in food.

Social information is important in many different contexts for vertebrate animals, from reproduction to discovery of novel food items to alarm signalling when predators are near [10,12]. This study demonstrates a novel benefit of public information in the context of resource limitation and finds that advance warning about declining food can lead to better outcomes during times of scarcity. If individuals in flocks struggle to meet energy demand due to poor foraging performance, declining food or most likely as an interaction between the two, they may serve as a harbinger of risk to those with higher foraging performance. Such benefits may in part underlie the social nature of songbirds that tend to form large flocks during times of increased risk of food scarcity in winter.

## Data Availability

Data have been provided in the Dryad Digital Repository [65]. Electronic supplementary material is available online [66].
